# Basal Implant Placement in the Anterior Aesthetic Zone: A Case Report

**DOI:** 10.7759/cureus.61782

**Published:** 2024-06-06

**Authors:** Ankita Pathak, Mithilesh M Dhamande, Smruti Gujjelwar, Prasanna R Sonar, Shubham U Tawade, Aashish Gupta

**Affiliations:** 1 Prosthodontics, Sharad Pawar Dental College and Hospital, Datta Meghe Institute of Higher Education and Research, Wardha, IND; 2 Oral Medicine and Radiology, Sharad Pawar Dental College and Hospital, Datta Meghe Institute of Higher Education and Research, Wardha, IND

**Keywords:** implants, bone density, esthetics, single piece implant, basal implant

## Abstract

Aesthetics are one of the primary goals of restorative care. Teeth that are traumatized in the anterior maxilla usually avulse or require extraction due to fractures. Rehabilitation is challenging in such a therapeutic state since it presents several anatomical and aesthetic issues. There are circumstances in which traditional implant placement is problematic. There must be enough bone for implant placement to be uneventful and successful. Other surgical therapies may be necessary in addition to implant placement for certain operations, such as extensive grafting, direct or indirect sinus lifts, and nerve lateralization. Certain procedures are required for these operations but are not always achievable. Because single-piece basal implants provide immediate temporization and loading while receiving adequate anchoring from the basal cortical bone, they have been extensively used to rehabilitate resorbed ridges. This case report demonstrates the placement of the basal implant in the anterior zone.

## Introduction

The anterior maxilla is crucial in aesthetics and function, making it particularly significant when considering dental trauma and its long-term effects. Consequently, any trauma or damage to this region can have profound psychological and social implications, impacting self-esteem and confidence. When it comes to childhood trauma, the anterior maxilla is the most affected [1]. With excellent long-term outcomes, implant-supported prostheses are now routinely used to rehabilitate partly or completely edentulous patients. The implant must be positioned correctly for the best functional and aesthetic outcomes, which may require sufficient alveolar bone and surrounding soft tissue. When this is deficient due to atrophy (range of remaining bone approximately 3-4 mm), consequences of periodontal disease, traumas, or congenital deformities, bone-grafting procedures, guided bone regeneration, and alveolar bone expansion are all possibilities to restore additional bone volume and keratinized mucosa. However, there are certain circumstances in which this bone grafting is not recommended [2].

Dr. Jean-Marc Julliet was the first to employ single-piece implants in 1972. The first person to create the "diskimplants" single-piece implant system was Dr. Gerard Scortecci. Later, basal osseointegrated implants (BOIs) were developed by Dr. Stefan Idhe, who was a pioneer in this field. These implants depend upon basal bone, which is less prone to infection and resorption, as an anchorage, as opposed to conventional implants, which use the cortical bone. Furthermore, additional surgical procedures for augmentation may be necessary for conventional implants, increasing the expense and length of treatment [3].

The jawbone is classified into two primary components in basal implantology: the basal bone and the tooth-bearing alveolus, also known as the crestal portion. The tooth-supporting crestal bone is less dense and more vulnerable to infection as a result of dental diseases, trauma, or medical procedures [3]. It is hence more prone to resorption. However, the basal bone has a thick, hard outer layer due to its heavy cortication. Resorption or infections hardly ever harm this dense cortical bone. Implants are well supported by the basal bone due to its structural characteristics. It is the perfect foundation for dental implants because of its density and resistance to biological decay [4].

Implant placement is a routine process in implant dentistry. Nonetheless, there are circumstances in which traditional implant placement is impractical. There must be enough bone for implant implantation to go smoothly and successfully (at least 13-15 mm in length and 5-7 mm in width). Other surgical therapies may be necessary in addition to implant implantation for certain operations, such as extensive grafting, direct or indirect sinus lifts, and nerve lateralization. These procedures are not always feasible and rely on certain methods [4,5].

To address all the drawbacks, new treatment techniques have been developed, such as basal implants. Basal implants are specially designed to attach to the basal cortical bone [6,7]. This case report depicts an immediate replacement of missing teeth in the anterior region with basal implants.

## Case presentation

**Figure 1:**
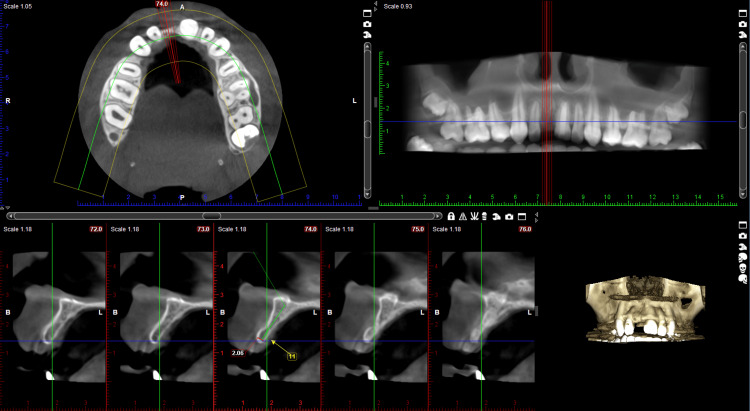
Preoperative CBCT showing missing tooth in the 11th region CBCT: Cone beam computed tomography Available buccolingual dimensions were 2.06 mm, and the apico-coronal dimension was 13 mm

**Figure 2:**
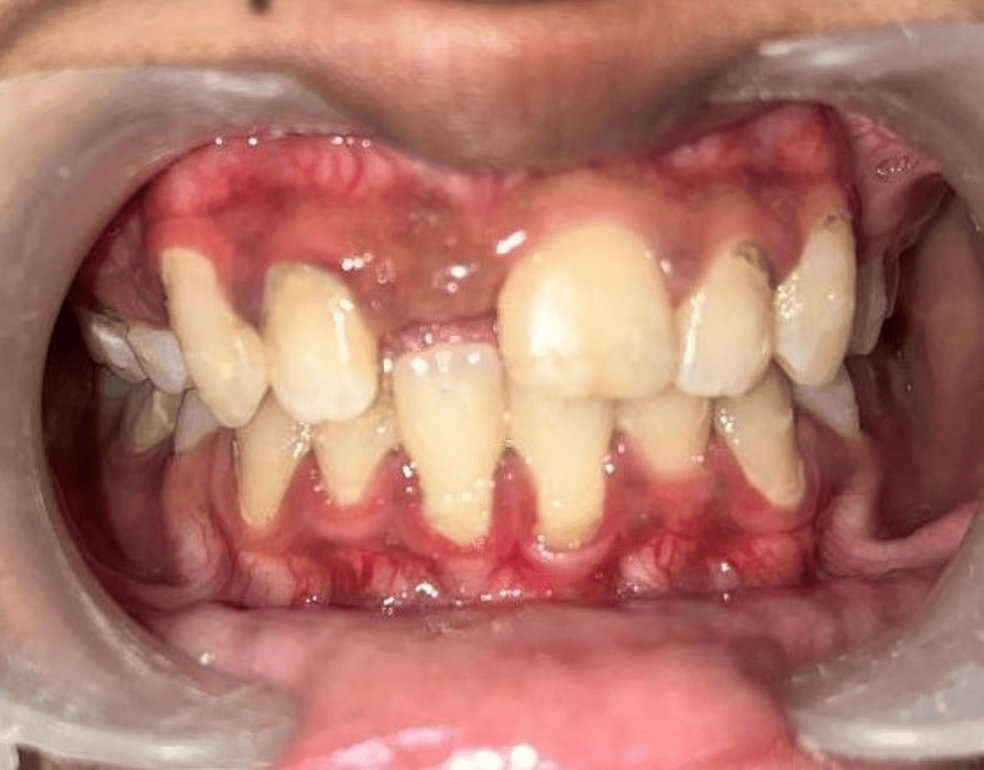
Preoperative clinical view showing the missing 11th tooth

A 19-year-old female patient presented to the Department of Prosthodontics with a chief complaint of missing anterior teeth in the 11th region and expressed a desire to have them replaced. The patient disclosed a history of trauma resulting from a road traffic accident (RTA) three months prior and expressed urgency in replacing the missing tooth. Intraoral examination revealed the absence of teeth in the 11th region. Various treatment options were discussed with the patient. There was a horizontal-type ridge defect at the affected site. A radiographic examination with cone beam computed tomography (CBCT) and clinical examination were performed, and the results are depicted in Figure 1 and Figure 2. 

Upon evaluation of the CBCT, it was noted that the buccolingual width in the 11 region was approximately 2 mm. Conventional endosteal implants typically require a minimum bone width of 5 mm, leaving 1 mm of bone around the implant post-placement. The option of an autogenous chin graft was considered; however, due to the patient's immediate need for tooth replacement, both possibilities were ruled out. With the patient's consent, it was decided to place a single-piece basal implant in the 11th region with bicortical engagement.

**Figure 3:**
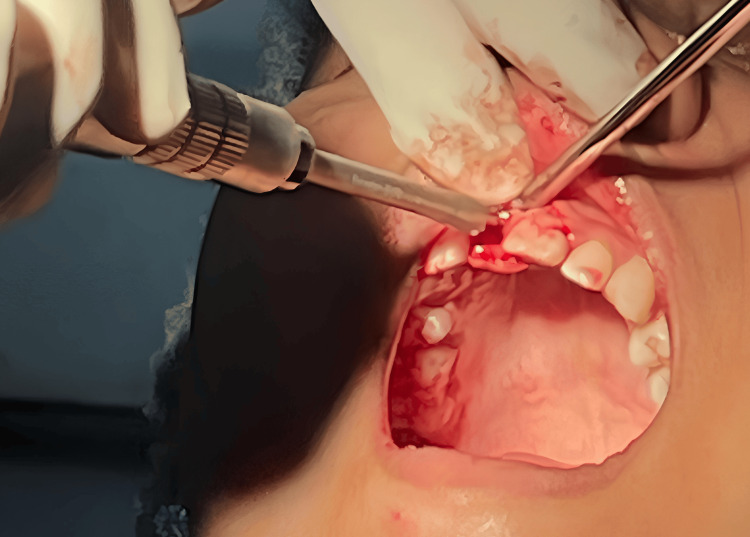
Orientation of basal implant at modified angulation A 20-degree angulation was provided to orient the basal implant in arch

**Figure 4:**
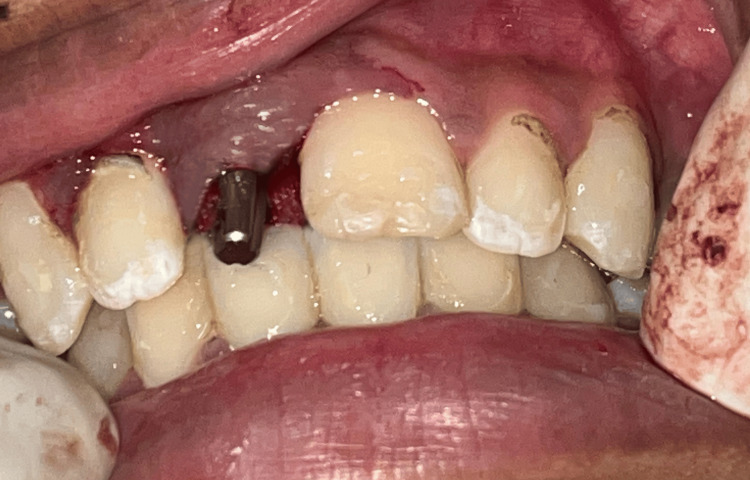
Basal implant in the 11th region. By using flapless technique, a basal implant was placed in the 11th region

Under all aseptic precautions and infraorbital nerve block, local anesthesia was administered in the 11th region. As it is the flapless technique, a bicortical screw (BCS) 2.7 x 13 mm basal implant was placed, and an angle correction was performed as necessary at the time of placement as shown in Figure 3. Figure 4 depicts basal implant in the 11th region.

**Figure 5:**
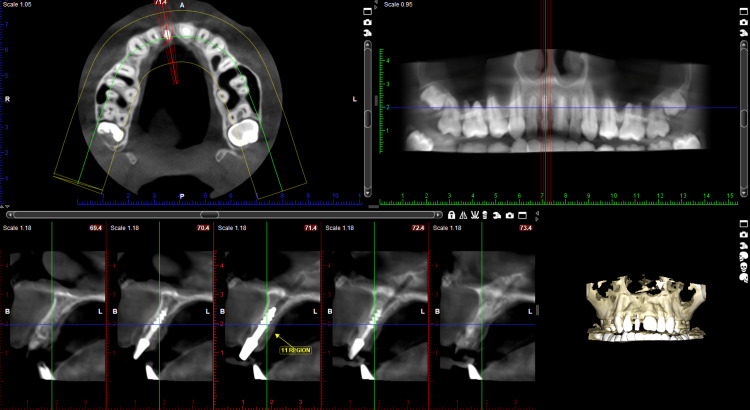
Postoperative CBCT showing the basal implant in the 11th region CBCT: Cone beam computed tomography Buccal and lingual cortical engagement seen in CBCT with basal implant in the 11th region

Postoperative CBCT confirmed the placement of the basal implant in the 11th region, as shown in Figure 5.

**Figure 6:**
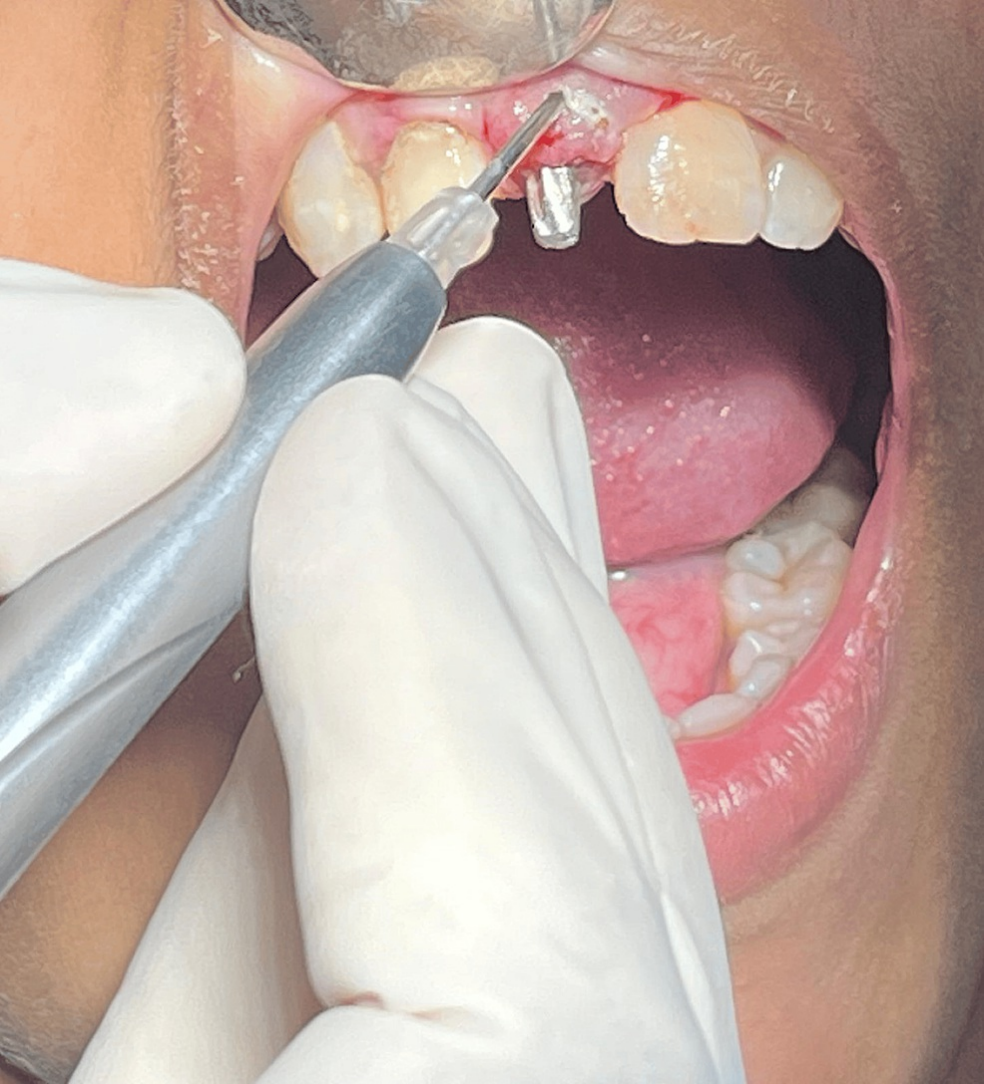
Laser gingivectomy performed to contour cervical gingiva

Following implant placement, a laser gingivectomy was performed to modify the gingival contour. Abutments were prepared, immediate loading was performed, and temporization using Luxatemp material was completed as shown in Figure 6.

**Figure 7:**
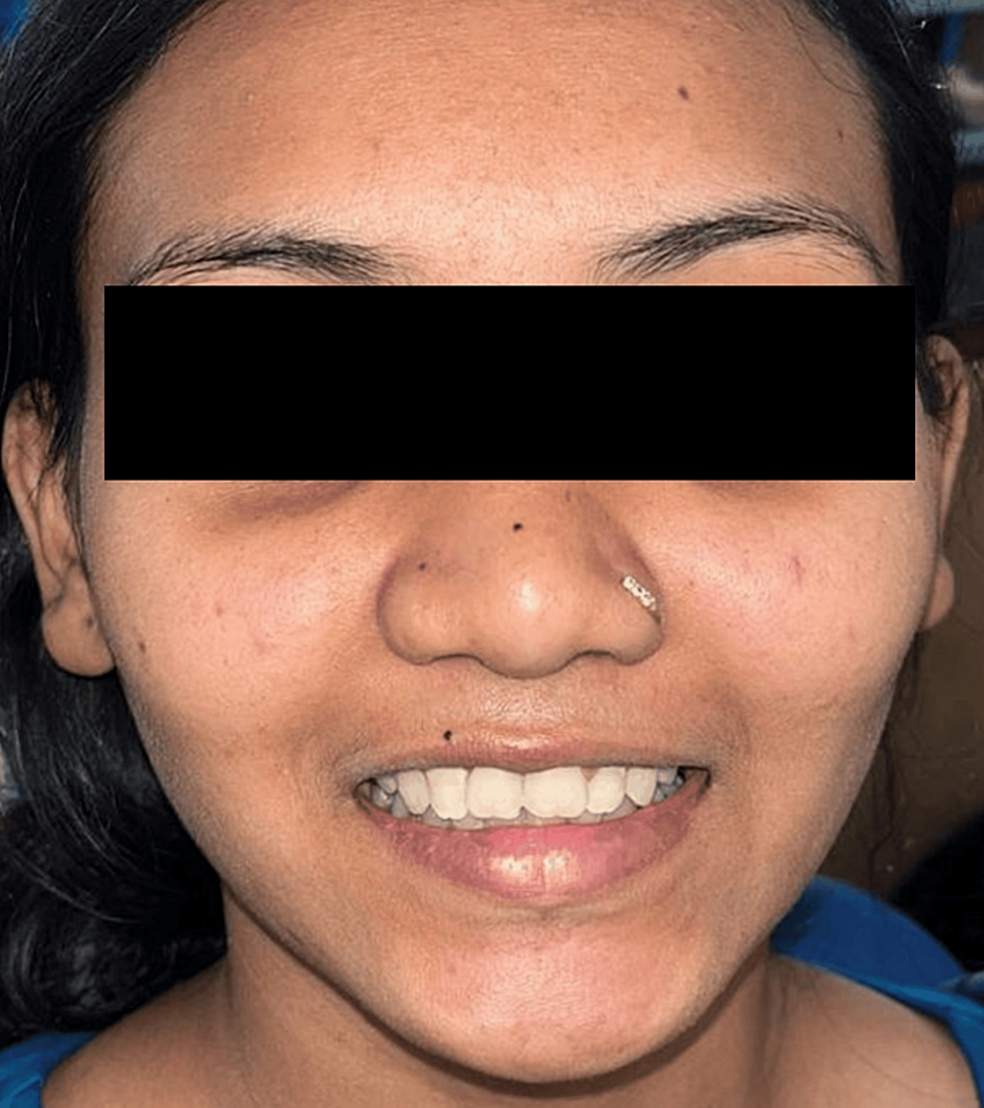
Cementation of the crown with a basal implant in the 11th region

The patient was followed up after three months, and a final impression was made using an addition silicon light body and putty. A metal-ceramic crown was fabricated for implantation in the 11th region, ensuring proper fit and aesthetics. Type 1 luting glass ionomer cement was used for cementation. The patient was provided with post-insertion care instructions and a follow-up program. At three-month follow-up appointment, the prosthesis was satisfactory in terms of aesthetics, patient acceptability, and gingival margin position. Figure 7 shows final prosthesis cementation.

## Discussion

The primary goals of prosthetic rehabilitation are to prevent overload osteolysis, to achieve excellent aesthetics, and to facilitate good hygiene practices. Considering they don't require significant augmentation, the research and development that these implants have undergone have made them a feasible choice for recovering atrophied jaws. They enable immediate loading. They can also be coupled with any implant and inserted using a flapless method [1,4].

In a one-piece implant design, the implant and its abutment combine to form a single unit, thereby closing off minute gaps between the implant and abutment. With all of these advancements in modern dentistry, the focus is now on shortening the duration of implant therapy. Three strategies have been devised to achieve this goal: immediate, early, and delayed loading [8,9]. The patient can begin using the prosthesis right away following implant placement owing to the immediate loading of the implants, which also reduces the duration of the overall treatment.

The type of flap chosen for the surgical operation is an additional essential consideration for one-piece implant insertion. Implant placement access has typically been obtained through a flap. However, research indicates that flap reflection frequently causes bone resorption surrounding natural teeth, and this also applies to implants [10]. The use of the flapless procedure to insert implants has grown in favor recently for several reasons, including less postoperative discomfort, reduced bleeding at the surgical site, elimination of the need for sutures, quicker recovery, and shorter surgical times [11].

The concept of basal implants is novel. According to numerous consensus findings on basal implantology, the theory of osseofixation, immediate loading, and implant of the site have all shown excellent results [3,5]. Numerous investigations have been conducted since then to compile information on its success and survival rates. Reports of patients with a follow-up time of one to five years have notably shown enhanced survival rates, despite the lack of large-scale investigations in this area. The survival of basal implants and endosseous implants was studied by Garg et al., who reported positive outcomes for a total of 52 implants: 34 endosseous and 18 basal [12].

One-piece implants have shown promise for replacing teeth in the aesthetic zone and posterior region. They have also shown long-term outcomes, lasting up to 10 years, indicating that it is possible to maintain the biological width and crestal bone level for periodontal stability [13,14].

Dental implants known as "basal implants" utilize the extremely dense cortical bone, which has the lowest tendency to resorb to support the implant. These implants are unique in that they are designed to attach to the basal cortical bone. The current basal implant is a prosthetic-driven system with an enhanced design and surgical protocol. Because of these qualities, several professionals worldwide use basal implantology in their practices, and thus far, these implants have produced incredibly positive outcomes [15]. To retain these forefront implants, basal implantology emerged, which comprises implant placement in the basal cortical bone, offering superior-quality cortical bones. Because they adhere to the guidelines of orthopedic surgery, they are also referred to as cortical, bicortical, and orthopedic implants [16,17]. They are known as disc or lateral implants as well. In densely packed native bone, basal implants offer multi-cortical support, ensuring absolute primary implant stability. Even though basal implants may compromise aesthetics in single-tooth replacement, they are a blessing for inadequate bone width and atrophied ridges [18,19].

Rehabilitation using implant-supported prostheses for partially and completely edentulous patients is widely accepted. Conventional Branemark systems require a 4-6 month wait before loading the implants, leaving patients without teeth or with a temporary removable prosthesis, which often discourages them from this option. To address these issues, basal implants were developed [3].

Basal implants are single-section implants where the implant body and abutment are fused, eliminating the connection interface complications seen in traditional two- and three-piece implants. They consist of three parts: the body, neck, and surface. Basal implants feature a design that addresses the complications of traditional two- and three-piece implants by integrating the implant body and abutment into a single unit. This single-section design eliminates issues at the connection interfaces and consists of three main components: the body, neck, and surface [3,4].

The implant body is characterized by its thin structure with wide threads. This design significantly increases the mechanical contact area between the implant and the bone, enhancing stability. Additionally, the wide threads improve vascularity around the implant, promoting better integration and healing. The implant neck serves as the connection between the implant and the abutment. Depending on the length of the implant, the abutment at the neck can be inclined from 15 to 25 degrees [20,21]. This inclination helps in optimizing the fit and orientation of the implant within the bone structure. The implant surface is typically polished to provide a smooth finish. This polished surface plays a crucial role in protecting the implant neck and body from bacterial and plaque attachment, reducing the risk of infection and ensuring the long-term success of the implant.

Implants placed in the basal bone can be loaded with teeth immediately because this bone is very strong, does not resorb over time, and supports stress well [4]. Since the cortical walls around the extraction site remain stable at the time of extraction, implants placed in fresh extraction sockets have a higher success rate than those placed after a delay [3,4].

There are two methods for the immediate loading of dental implants. The first method uses a compression screw, while the second relies on the cortical anchorage of thin screw implants (BCS). Conventional crestal implants are suitable when there is adequate vertical bone [22,23]. They work well when sufficient bone is present initially, but their prognosis declines with augmentation procedures, which increase risks, costs, and the number of required operations. Patients with severely atrophied jaw bones often receive little or no treatment when crestal implants are the first choice. Basal implants offer an effective alternative, particularly for patients lacking sufficient vertical bone or those needing immediate implant loading [3-5].

## Conclusions

In conclusion, the case report presented herein demonstrates the successful placement and rehabilitation of a missing anterior tooth utilizing a basal implant in a patient with insufficient buccolingual bone breadth. Traditional implant placement techniques often pose challenges in cases of bone deficiency, trauma, or immediate need for restoration. However, with the advent of basal implants, such limitations are addressed effectively, providing immediate temporization and loading while ensuring adequate anchoring from the basal cortical bone. Despite being less documented in the anterior aesthetic zone, the efficacy of basal implants in such scenarios is highlighted in this report. The utilization of flapless techniques further enhances patient outcomes by reducing postoperative discomfort, bleeding, and surgical times. With advancements in implant dentistry focusing on immediate loading strategies and prosthetic-driven systems, basal implants offer a promising solution for cases where conventional methods may not suffice. Continued research and documentation are essential to further validate the success and longevity of basal implants in dental rehabilitation, particularly in challenging anatomical and aesthetic contexts.
